# Are urgent care centers a viable venue for recruitment in clinical trials?

**DOI:** 10.1186/s13063-015-1074-6

**Published:** 2015-12-01

**Authors:** Hersh V. Goel, Trevor A. Jones, David P. Skinner

**Affiliations:** Johns Hopkins Bayview Medical Center, 4940 Eastern Ave., Baltimore, MD 21224 USA; Southern Arizona Urgent Care, LLC, 7725 N Oracle Rd #131, Oro Valley, AZ 85704 USA; PharmTrials, LLC, 7725 N Oracle Rd #121, Oro Valley, AZ 85704 USA

**Keywords:** Urgent care, Recruitment, Clinical trial, Emergency care

## Abstract

Clinical trials are extremely difficult to undertake and recruiting patients for these trials is one of the most significant challenges. This brief report sought to determine the suitability of an urgent care center as a research recruitment site by assessing its patients’ views on participating in clinical trials. One thousand and two subjects were surveyed over a 45-day period; 9.0 % had previously participated in clinical trials while 46.6 % indicated they would be interested in participating in future clinical trials if given the opportunity. This research suggests that the urgent care venue is a viable recruitment source for clinical trials.

**Electronic supplementary material:**

The online version of this article (doi:10.1186/s13063-015-1074-6) contains supplementary material, which is available to authorized users.

## Findings

The first randomized controlled trial of a medical treatment was performed in 1947 [1], and since then clinical trials have been instrumental in advancing discoveries in medicine. Successfully conducting clinical trials, however, is no easy task. Study participant recruitment is becoming increasingly difficult and it has been suggested that patients are now less willing to participate in clinical trials than ever before [2]. With the burgeoning of urgent care medicine, “the provision of immediate outpatient medical service for the treatment of acute and chronic illness and injury”, [3] the question arises: can the urgent care model be a mode for study participant recruitment? Given the episodic nature of this care, it is easy to assume the answer is “no.” In fact, however, this question has been heretofore unexplored in the medical literature. Americans receive most of their healthcare in ambulatory care settings, [4] and increasingly in the over 6,400 [5] urgent care centers across the nation. The purpose of this brief report is to understand urgent care patients’ perspectives on clinical trial involvement in an episodic care-based framework, the answer of which could help to shape the future of study participant recruitment.

One thousand and two subjects (74.2 % response rate) completed an electronic Patients seeking medical care were provided a three minute seven item surveysurvey between 14 April and 28 May 2015 at an urgent care unit housed within a fully integrated ambulatory medical center in Southern Arizona. The ambulatory medical center serves the full primary care continuum and includes extensive point of care testing, in-house diagnostic imaging capabilities, and an in-house pharmacy. Patients seeking medical care were provided a 3-minute 7-item survey (Additional file 1) on an Internet-enabled electronic tablet. Medical assistants offered patients the option of completing the voluntary survey after they were triaged and during the standard waiting period prior to being seen by the healthcare provider. No identifiable information was collected. Patients under 18 years of age and those with intellectual disabilities were excluded from participation. The study protocol was reviewed and approved by IntegReview Institutional Review Board.

Subjects were stratified into those who had previously participated in clinical trials (Experienced Group) and those who had not (Unexperienced Group). Pearson’s chi-squared testing was performed to determine differences in survey responses between the two groups. Analyses were performed using STATA/SE 12.1 (StataCorp LP, College Station, TX, USA).

Only 9.0 % of subjects had previously participated in clinical trials (Table 1) while 46.6 % of all subjects indicated that they would be interested in participating in future clinical trials that could benefit them (Table 2). An additional one third of subjects (33.7 %) said they were unsure of whether they would participate in such clinical research, while only 19.7 % declined (Table 2). Subjects who had previously participated in clinical trials were 12.3 % more likely to want to participate in future research compared to their counterparts (χ^2^[6] = 7.41, *P* = 0.025, Table 2). Furthermore, they were 61.4 % more likely to want to advance medicine as a motivation for participating in clinical trials, while unexperienced subjects were over 3 times more likely to be motivated by the possibility of earning free medical care (χ^2^[6] = 15.93, *P* = 0.014, Table 3). Greater than 50% of patients were either unsure as to why they would not participate in future clinical trials, feared being a "guinea pig" or felt they did not have enough information about available studies (Table 4). Subjects trusted physicians the most (40.3 %) as their preferred source to learn more about clinical trial opportunities (Table 5). Subjects answered each question at a 100 % response rate.

**Table 1:** Participation in previous clinical trials (*n* = 1002)

No (Unexperienced Group)	Yes (Experienced Group)
91.0 % (912)	9.0 % (90)

**Table 2:** Willingness to participate in future clinical trials stratified by previous participation (*n* = 1002)

	Unexperienced Group^a^	Experienced Group^a^	Overall
No	20.6 % (188)	10.0 % (9)	19.7 % (197)
Unsure	33.9 % (309)	32.2 % (29)	33.7 % (338)
Yes	45.5 % (415)	57.8 % (52)	46.6 % (467)

^a^A statistically significant difference exists between the Unexperienced and Experienced Groups (χ^2^[6] = 7.41, *P* = 0.025)

**Table 3:** Primary reason for participation in future clinical trials stratified by previous participation (*n* = 1002)

	Unexperienced Group^a^	Experienced Group^a^	Overall
Advance medicine	18.4 % (168)	30.0 % (27)	19.5 % (195)
Earn extra money	5.3 % (48)	8.9 % (8)	5.6 % (56)
Improve my condition	27.4 % (250)	28.9 % (26)	27.5 % (276)
Improve the lives of others	22.7 % (207)	21.1 % (19)	22.6 % (226)
Free medical care	3.6 % (33)	1.1 % (1)	3.4 % (34)
Other	2.9 % (26)	0.0 % (0)	2.6 % (26)
Unsure	19.7 % (180)	10.0 % (9)	18.9 % (189)

^a^A statistically significant difference exists between the Unexperienced and Experienced Groups (χ^2^[6] = 15.93, *P* = 0.014)

**Table 4:** Primary reason for non-participation in future clinical trials stratified by previous participation (*n* = 1002)

	Unexperienced Group^a^	Experienced Group^a^	Overall
Distance/travel time burden	10.4 % (95)	21.1 % (19)	11.4 % (114)
Fear of receiving placebo	8.3 % (76)	8.9 % (8)	8.4 % (84)
Health insurance will not pay	9.7 % (88)	8.9 % (8)	9.6 % (96)
Lack of information about available studies	19.1 % (174)	15.6 % (14)	18.8 % (188)
New treatment is not better than standard	5.9 % (54)	5.6 % (5)	5.9 % (59)
Fear of being a “guinea pig”	16.1 % (147)	12.1 % (11)	15.8 % (158)
Other	6.5 % (59)	11.1 % (10)	6.9 % (69)
Unsure	24.0 % (219)	16.7 % (15)	23.4 % (234)

^a^A statistically significant difference exists between the Unexperienced and Experienced Groups (χ^2^[7] = 14.12, *P* = 0.049)

**Table 5:** Trusted sources to learn about clinical trials (*n* = 1002)

	Unexperienced Group^a^	Experienced Group^a^	Overall
Government agency	5.7 % (52)	14.4 % (13)	6.5 % (65)
Non-profit group	9.2 % (84)	8.9 % (8)	9.2 % (92)
Patients who previously participated in clinical trials	18.1 % (165)	14.4 % (13)	17.8 % (178)
Pharmaceutical company	1.1 % (10)	0.0 % (0)	1.0 % (10)
Physicians that have conducted clinical trials	40.1 % (366)	42.2 % (38)	40.3 % (404)
Other	2.9 % (26)	2.2 % (2)	2.8 % (28)
Unsure	22.9 % (209)	17.8 % (16)	22.5 % (225)

^a^A statistically significant difference does not exist between the Unexperienced and Experienced Groups (χ^2^[6] = 12.43, *P* = 0.053)

## Discussion

This study demonstrates a clear interest amongst urgent care patients at an integrated ambulatory medical center to participate in clinical trials. This could have significant implications for the recruitment of participants in future clinical trials, an area that is already burdened by many impediments [6]. Despite their growth, urgent care centers have not been previously utilized in this capacity and can potentially aid in filling the recruitment gap. This study also identifies differences between those who have previously participated in clinical trials and those who have not; the former was most motivated by the prospect of helping to advance medicine while the latter by receiving free medical care. Future studies should explore the reasons for this finding. This study is limited by the hypothetical nature of its findings and that it was conducted at only one integrated ambulatory medical center. Additional research must be conducted to determine whether individuals actually do participate in clinical trials and research should be pursued at more organizations to improve generalizability.

## Additional file

Additional file 1:
**Clinical trial survey.** This is the actual survey that was provided to each subject. (DOCX 17 kb)

## References

[1] Miller FG (2014). Clinical research before informed consent. Kennedy Inst Ethics J.

[2] Cox K, McGarry J (2003). Why patients don’t take part in cancer clinical trials: an overview of the literature. Eur J Cancer Care.

[3] AAUCM. What is Urgent Care? http://aaucm.org/about/urgentcare/default.aspx. Accessed 2 November 2015.

[4] Green LA, Fryer GE, Yawn BP, Lanier D, Dovey SM (2001). The ecology of medical care revisited. N Engl J Med.

[5] America UCAo. Industry FAQs. http://www.ucaoa.org/?page=IndustryFAQs&hhSearchTerms=%22faq%22. Accessed 14 March 2015.

[6] Lovato LC, Hill K, Hertert S, Hunninghake DB, Probstfield JL (1997). Recruitment for controlled clinical trials: literature summary and annotated bibliography. Control Clin Trials.

