# Supplementary material for: Are urgent care centers a viable venue for recruitment in clinical trials?
Source: Trials. 2015 Dec 1;16:543. doi: 10.1186/s13063-015-1074-6 (PMC4665919; doi:10.1186/s13063-015-1074-6)
Supplement: Additional file 1: — Clinical trial survey. This is the actual survey that was provided to each subject. (DOCX 17 kb) [file 13063_2015_1074_MOESM1_ESM.docx]

**Additional file 1**

Clinical Trial Survey:

1. Have you previously participated in a clinical trial? Note: A clinical trial involves research using human volunteers that is intended to add to medical knowledge. More information can be found here: https://clinicaltrials.gov/ct2/about- studies/learn
   1. Yes
   2. No
2. How did you hear about the clinical trial?
   1. Online/Internet
   2. Media
   3. Advertisement
   4. Physician or other medical provider
   5. Patient organization
   6. Unsure
   7. N/A - I have not previously participated in a clinical trial
   8. Other
3. Participating in the clinical trial was beneficial to you
   1. Strongly Agree
   2. Agree
   3. Neutral
   4. Disagree
   5. Strongly Disagree
   6. N/A - I have not previously participated in a clinical trial
4. If given the opportunity to participate in a new clinical trial that seeks to treat your condition/illness, would you?
   1. Yes
   2. No
   3. Unsure
5. What is the primary reason you WOULD participate? If you answered "No" to Question #4, what might convince you to participate?
   1. Advance medicine
   2. Improve the lives of others
   3. Improve my condition
   4. Earn extra money
   5. Receive free medical care
   6. Unsure
   7. Other
6. What is the primary reason you would NOT participate? If you answered "Yes" to Question #4, what might discourage you from participating?
   1. New treatment proposed by study protocol is not better than the standard treatment
   2. Fear of receiving placebo
   3. Fear of being treated like a "guinea pig"
   4. Distance/travel time to participate in study is too great
   5. Health insurance will not pay
   6. Lack of information about available studies
   7. Unsure
   8. Other
7. Which of the following sources do you trust to learn more about clinical trials?
   1. Non-profit advocacy group
   2. Patients who previously participated in clinical trials
   3. Physicians that have conducted clinical trials
   4. Government agency (such as National Institutes of Health)
   5. Pharmaceutical company
   6. Unsure
   7. Other
